# APC/C: current understanding and future perspectives

**DOI:** 10.12688/f1000research.18582.1

**Published:** 2019-05-23

**Authors:** Hiroyuki Yamano

**Affiliations:** 1 Cell Cycle Control Group, UCL Cancer Institute, University College London, Paul O’Gorman Building, 72 Huntley Street, London, WC1E 6DD, UK

**Keywords:** anaphase-promoting complex/cyclosome (APC/C), cyclin-dependent kinase 1 (Cdk1), cell cycle, proteolysis, ubiquitin

## Abstract

The separation of sister chromatids at anaphase, which is regulated by an E3 ubiquitin ligase called the anaphase-promoting complex/cyclosome (APC/C), is arguably the most important irrevocable event during the cell cycle. The APC/C and cyclin-dependent kinase 1 (Cdk1) are just two of the many significant cell cycle regulators and exert control through ubiquitylation and phosphorylation, respectively. The temporal and spatial regulation of the APC/C is achieved by multiple mechanisms, including phosphorylation, interaction with the structurally related co-activators Cdc20 and Cdh1, loading of distinct E2 ubiquitin-conjugating enzymes, binding with inhibitors and differential affinities for various substrates. Since the discovery of APC/C 25 years ago, intensive studies have uncovered many aspects of APC/C regulation, but we are still far from a full understanding of this important cellular machinery. Recent high-resolution cryogenic electron microscopy analysis and reconstitution of the APC/C have greatly advanced our understanding of molecular mechanisms underpinning the enzymatic properties of APC/C. In this review, we will examine the historical background and current understanding of APC/C regulation.

## Introduction

The ubiquitin pathway is an ATP-dependent tagging system which regulates a plethora of events in eukaryotic cells by controlling protein stability, localisation, assembly or activity of the target substrate ^1– 3^. Together with phosphorylation, the ubiquitin-tagging “ubiquitylation” is the most frequently observed post-translational modification *in vivo*. Thus, it is no exaggeration to say that at least some key proteins in many seminal pathways and signalling events observed in our body are regulated by ubiquitylation. In this process, ubiquitin, a highly conserved 76-residue protein, is initially linked to a ubiquitin-activating enzyme (E1) in a reaction that uses ATP. The activated ubiquitin is then transferred to a small ubiquitin-conjugating enzyme (E2), forming a thioester-linked E2-ubiquitin intermediate (E2~Ub). E2 acts either alone or in conjunction with an E3 ubiquitin ligase to conjugate ubiquitin, most commonly, onto the ε-amino group of lysine residues in substrate proteins, forming an isopeptide bond ^1, 2, 4^. These seemingly simple sequential actions of three enzymes (E1-E2-E3) are tightly controlled to achieve accurate and appropriately timed ubiquitylation/proteolysis. More than 3% of genes in eukaryotic genomes are involved in the ubiquitin system, using multiple layers of regulation, to maintain homeostasis throughout the cell and organism.

The anaphase-promoting complex/cyclosome (APC/C) was discovered as an unusually large E3 ubiquitin ligase of cyclin B by biochemical fractionation of *Xenopus* egg and clam oocyte extracts ^5, 6^. Around the same time, genetic screening using yeast mutants defective in cyclin B proteolysis during anaphase and G _1_ identified genes such as *APC6/CDC16* and *APC8/CDC23* ^7^. Purification of APC/C from *Xenopus* egg extract demonstrated the presence of homologues of budding yeast Apc6/Cdc16 and Apc3/Cdc27, which were required for cyclin destruction and anaphase progression in fungi and mammalian cells ^8– 11^. Hence, the idea that the APC/C ubiquitin system ^5– 7^, essential cellular machinery, controls not only cyclin destruction but also the initiation of anaphase in all eukaryotes arose and turned out to be true. Shortly thereafter, securin/Cut2/Pds1 was identified as the first non-cyclin APC/C substrate required for sister chromatid separation ^12, 13^. This opened up a new chapter of proteolysis-driven cell cycle control in the mid-1990s, and to date a considerable number of APC/C substrates have been identified.

APC/C activity is under tight control to ensure that APC/C substrates are ubiquitylated and degraded at the right time and the right place during the cell cycle ^14– 18^. What are the underlying mechanisms? How can we control it if it is mis-regulated? Although we have known for a quarter of a century that the APC/C is an E3 ubiquitin ligase, the enormity (1.2 MDa) and complexity (14 subunits) of the enzyme have hindered the reconstitution of apo-APC/C complex and subsequent detailed analysis until recently ^19^. Now, high-resolution structural studies using reconstituted APC/C and multidisciplinary approaches have advanced our understanding of the APC/C. Here, we give an overview of APC/C regulation to date and highlight emerging themes. Readers interested in aspects of APC/C structural regulation that are beyond the scope of this review are pointed to recent comprehensive review articles ^20, 21^.

## The APC/C is a multi-subunit cullin-RING E3 ubiquitin ligase

**Figure 1.**
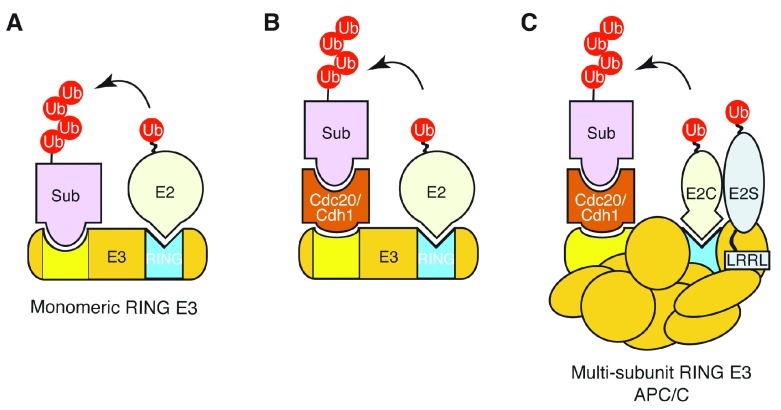
Schematic diagrams of RING E3 ubiquitin ligases. RING-type E3 ligases serve as scaffolds to bring together the E2~Ub conjugate and the substrate. E3s play a role in stimulating Ub transfer to the substrate from E2~Ub conjugate. E2-binding RING domain is coloured in light blue. ( **A**) Monomeric RING E3 ubiquitin ligases (for example, c-Cbl). ( **B**) A simplified cartoon view of APC/C RING E3 ubiquitin ligase with a co-activator such as Cdc20 and Cdh1. ( **C**) The APC/C is a multi-subunit cullin-RING E3 ubiquitin ligase that uses two E2s. APC/C, anaphase-promoting complex/cyclosome.

The APC/C belongs to the RING finger family of E3 ubiquitin ligases ^22– 26^. Unlike the HECT E3s that form E3~Ub intermediates during ubiquitin transfer, the RING E3s lack active sites and do not participate chemically in ubiquitin transfer. Instead, the RING E3 ubiquitin ligase functions as a scaffold that brings together an E2~Ub and a substrate ( Figure 1A), thereby catalysing ubiquitin transfer from the E2 to the substrate. The E3s typically behave as two-substrate enzymes in which the E2~Ub and substrate are the two reactants whose binding affinities both influence the reaction rate. In addition, the APC/C exploits one more component, a co-activator such as Cdc20 and Cdh1, as a substrate recruitment adaptor and APC/C activator ( Figure 1B). Thus, APC/C activation can be regulated by multiple mechanisms, including the interactions or spatiotemporal regulations among these four elements together with ubiquitin molecules, all of which can be subject to post-translational modifications such as phosphorylation and inhibitor/pseudo-substrate binding. It is also likely that individual substrate–co-activator binding strength or mode or both regulate the formation of APC/C-E2~Ub and the substrate ubiquitylation. Adding yet another level of complexity, the APC/C (E3) consists of multiple subunits and exploits two E2 enzymes (for example, Ube2C and Ube2S) to achieve programmed ubiquitylation ( Figure 1C).

## Structure and mechanisms of the APC/C

**Figure 2.**
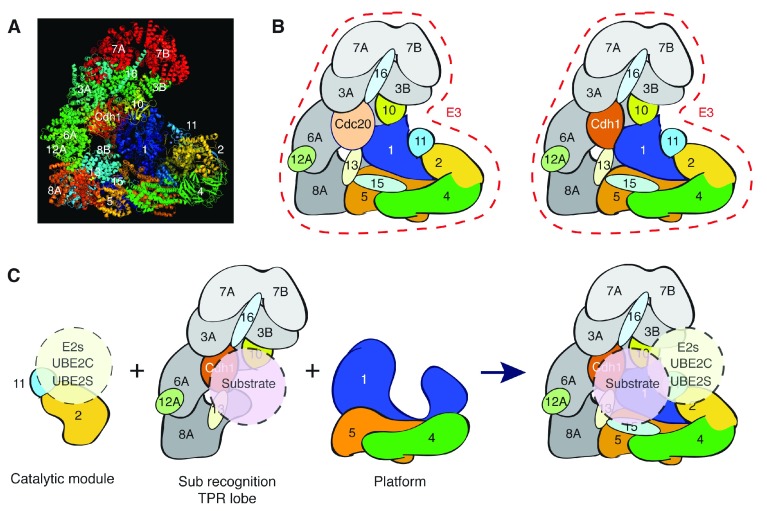
APC/C structure and overall organisation. ( **A**) APC/C structure. The image was generated by using the Protein Data Bank file (4UI9). The indicated numbers represent APC/C subunits. ( **B**) Schematic view of the APC/C structure based on ( **A**). Left: APC ^Cdc20^; right: APC/C ^Cdh1^. Cdc20 activates the APC/C in metaphase and anaphase to degrade substrates such as cyclin B and securin and then Cdh1 takes over to degrade APC/C substrates in late anaphase and G _1_. ( **C**) The APC/C complex can be divided into three modules: the catalytic module (Apc2-Apc11) that interacts with E2s, the substrate recognition TPR lobe and the scaffolding platform (Apc1-Apc4-Apc5). During the ubiquitylation catalysis, both substrate and E2s are positioned in or near the APC/C central cavity. APC/C, anaphase-promoting complex/cyclosome; TPR, tetratricopeptide repeat.

**Figure 3.**
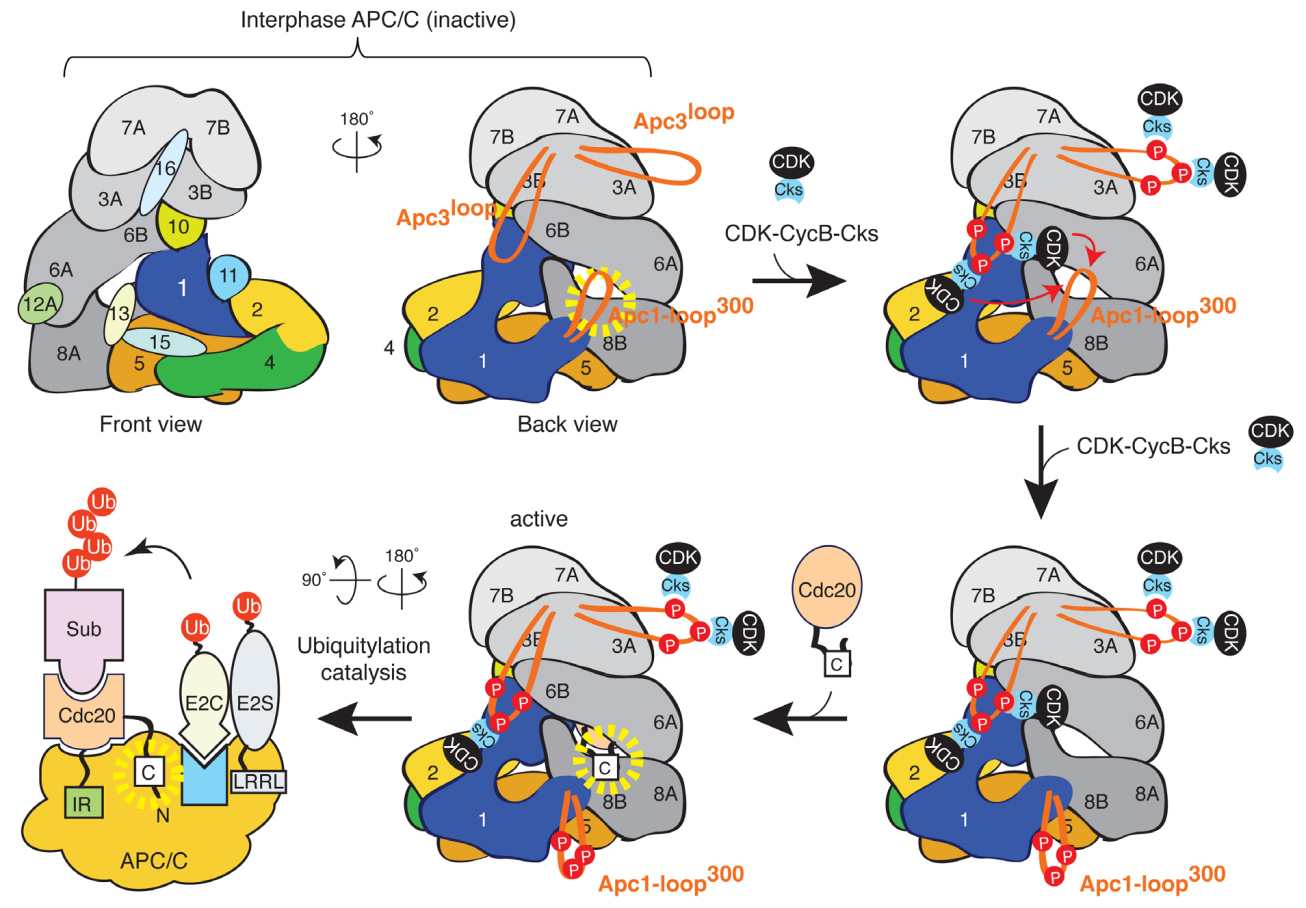
Phosphorylation-dependent activation of the APC/C for APC/C ^Cdc20^. Interphase APC/C is inactive without the recruitment of Cdc20, which is presented from a front view and a back view of the APC/C. The disordered loop domain of Apc1 (Apc1-loop ^300^), which is located in the N-terminal WD40 domain, blocks Cdc20-NTD access to the APC/C, in particular the C-box–binding sites on Apc8B. Yellow dotted circle highlights the C-box–binding site. In mitosis, Cdk1-cyclinB-Cks phosphorylates the disordered loop domain of Apc3 (Apc3 ^loop^), which allows Cks-bound Cdk1-cyclin B loading to Apc3 ^loop^. Cdk1-cyclinB-Cks then stimulates phosphorylation of Apc1-loop ^300^ as an intramolecular phosphorylation relay. Upon phosphorylation, inhibitory domain Apc1-loop ^300^ is dislocated from the C-box–binding site, allowing Cdc20 association, the C-box-dependent activation and subsequent ubiquitylation catalysis (“cartoon view of the APC/C”). The isoleucine-arginine (IR) tail of Cdc20 binds to Apc3 and the C-box interacts with Apc8B for activation of the APC/C. Both IR tail binding and C-box binding ensure stable binding of co-activator (Cdc20) to the APC/C. The WD40 domain of co-activator is responsible for substrate degron recognition. The RING subunit Apc11 is coloured in light blue. APC/C, anaphase-promoting complex/cyclosome.

Early cryogenic electron microscopy (cryo-EM) studies of yeast and vertebrate APC/C revealed that APC/C has a triangular or asymmetric heart-shape (V-shape) conformation ^27– 30^, which has been refined with the latest high-resolution cryo-EM ^31– 33^ ( Figure 2A). The APC/C complex consists of 14 mostly highly conserved subunits (Apc1–8, 10–13, 15 and 16) in metazoans (13 subunits in yeast) together with a structurally related interchangeable Cdc20/Fizzy family of co-activators such as Cdc20 and Cdh1, generating an “active” macromolecular machine exceeding 1.2 MDa ( Figure 2B). It should be noted that co-activators are not stoichiometric components of the APC/C, but the association of co-activators with the APC/C is essential for the APC/C to function. The most prevalent structural motif is a 34–amino acid tetratricopeptide repeat (TPR), which is present in five subunits (Apc3, Apc5, Apc6, Apc7 and Apc8), highlighting their role in coordinating the higher-order assembly and protein recognition/binding. As an organised structure, the APC/C complex can be divided into three sub-complex structures: the catalytic sub-complex (catalytic module), the substrate recognition sub-complex (TPR lobe) and the scaffolding sub-complex (platform) ( Figure 2C). The catalytic module consists of Apc11, the RING domain subunit and Apc2, the cullin subunit. The minimal module of Apc11-Apc2 (heterodimer) can catalyse ubiquitin transfer but with poor substrate specificity ^24– 26^. The substrate recognition TPR lobe comprises four TPR subunits (Apc7, Apc3, Apc6 and Apc8), each of which forms a V-shaped homodimer via N-terminal domains, which are packed in a parallel fashion resulting in the formation of a left-handed superhelical structure. Two copies of Apc12/Cdc26/Hcn1, which had been shown to genetically interact with Apc6/Cdc16/Cut9 ^34, 35^, stabilise Apc6A and Apc6B as molecular chaperones. Apc13 and Apc16 also help stabilise TPR subunit interaction and the assembly of the complex. Importantly, the substrate recognition of the TPR lobe is through the WD40 domain of Cdc20/Cdh1 and Apc10 ^36, 37^, both of which interact with the C-terminal TPR grooves of Apc3 through their C-terminal isoleucine-arginine (IR) tails. Finally, the scaffolding sub-complex of the APC/C comprises the platform subunits Apc4 and Apc5 (heterodimer) and the largest subunit Apc1, which bridges the catalytic module Apc11-Apc2 and the TPR lobe in catalytically favourable conformations ( Figure 2C). Since Apc4 and Apc5 had been shown to genetically interact with each other, the dimer formation had been suspected and the detailed interaction has been solved by high-resolution EM studies ^31, 32^. Apc1 has a WD40 beta-propeller domain containing several disordered loop domains at the N-terminus, one of which mediates phosphorylation-dependent APC/C control ( Figure 3). It also contains a central PC (proteasome-cyclosome) repeat domain, which interacts with Apc10, although the detailed regulation remains elusive. In the overall topology, the surprising beauty of the whole is that vital ubiquitylation elements such as a catalytic E2-binding module (Apc11-Apc2) and substrate-binding module (Cdc20/Cdh1 and Apc10) are all positioned facing a central cavity “catalytic centre” on this enormous multi-subunit complex ^31, 32^ ( Figure 2).

## The APC/C employs two E2s and assembles poly-ubiquitin chains

Ubiquitin has seven lysine residues (K6, K11, K27, K29, K33, K48 and K63), so eight structurally distinct types of poly-ubiquitin chain linkage can be formed, together with the N-terminal methionine (M1; a head-to-tail linear linkage). The anaphase-promoting complex (APC/C) can assemble K11-linked and K48-linked ubiquitin chains on substrates ^38– 45^. In order to build K11-linked ubiquitin chains, the metazoan APC/C uses two families of E2 enzymes: a “chain-initiating” E2 such as Ube2C/UbcH10 and Ube2D/UbcH5 and an “elongating” E2 such as Ube2S ^38– 40, 46, 47^. The APC/C facilitates these “team tagging” reactions by placing Ube2C and Ube2S at dedicated locations within the APC/C complex ^31, 32^. Ube2C binds the RING subunit Apc11 and Apc2 and transfers the first ubiquitin onto substrates (that is, multiple monoubiquitylation), whereas Ube2S binds non-RING subunits Apc2 and Apc4 through its C-terminal LRRL tail and elongates the K11-linked ubiquitin chains onto substrate-attached ubiquitin. RING subunit Apc11 involvement with Ube2C binding is as expected, but Apc2 plays an important role in interacting with both Ube2C and Ube2S through the winged-helix B (WHB) domain and the N-terminal domain of Apc2, respectively ^32, 48, 49^. It has also been reported that the back surface of Apc11 has an additional role in tracking and presenting the acceptor ubiquitin of the growing ubiquitin chain onto Ube2S, thereby ensuring K11-linked ubiquitin chain formation ^31, 50, 51^. Yet detailed mechanisms and the control of Ube2C and Ube2S loading and activity are mostly unknown. Interestingly, it has recently been observed that Ube2S does not simply extend a ubiquitin chain but creates mixed or branched K11/K48-linked ubiquitin chains, which act as better degradation signals for the proteasomal receptors than homotypic K11- or K48-linked ubiquitin chains ^41– 43^. How proteasomal ubiquitin-receptor proteins by themselves or in combination with UBL-UBA shuttle factors (for example, Rad23 and Dsk2) efficiently recognise branched ubiquitin chain configuration is not known. It is possible that mixed ubiquitin chains are more resistant to de-ubiquitylating enzymes (DUBs). In the past, E2 enzymes were considered just intermediates of the ubiquitin pathway, but more “active” roles have recently been discovered ^52, 53^. Not only “team tagging” but also new layers of E2 regulation might emerge in APC/C regulation in the future.

## Multifaceted regulation of the APC/C is mediated primarily by co-activators

Cdc20/Fizzy, a co-activator of the APC/C, was originally discovered as fly and yeast mitotic mutants that failed to initiate the onset of anaphase. Cdh1 was subsequently identified as a G _1_ co-activator ^54– 58^. Cdc20 or Cdh1 is around 55 kDa and constitutes less than 5% of the total mass of the APC/C complex (1.2 MDa), but the size does not matter. A co-activator has an absolute requirement for APC/C-dependent ubiquitylation. Initially, the WD40 domain-mediated “substrate capture” role was revealed ^59– 61^ and later the “activation role” through the C-box (a conserved motif in the Cdc20/Fizzy family of proteins) at the N-terminal domain was uncovered ^62, 63^. From biochemical and EM studies, the activation mechanism is thought to be through conformational changes within the APC/C complex; the C-box and Apc8B interaction shifts the catalytic module (Apc11-Apc2) upward and positions it in a catalytically favourable conformation, allowing E2~Ub loading ^32, 33, 63^. Here, three key facets of APC/C regulation via co-activators (that is, substrate recognition, phospho-regulation and inhibition) will be discussed briefly.

## Co-activator–substrate affinity might determine the rate of ubiquitylation

Substrate recognition, which is a prerequisite for ubiquitylation catalysis, is one of the most important roles performed by co-activators, together with Apc10 ^36, 37, 59– 61, 64, 65^. The APC/C substrates have a destruction motif or degron module sequence to be recognised by the WD40 domain of co-activators. The best-defined destruction motifs are the destruction box (D-box) with a consensus of RxxLxxxxN ^66^ and the KEN-box, named after its consensus sequence, KENxxxN ^67^, although the amino acid residues outside of the core RxxL and KEN are far more variable. The ABBA motif (Fx[ILV][FY]x[DE]) conserved in cyclin A, BubR1, Bub1 and Acm1 is a more recently characterised APC/C degron ^68– 70^. Also, there are less characterised degrons such as CRY-box or O-box and presumably as-yet-unidentified cryptic D-box or KEN-box exist. Each degron binds to a designated surface of the WD40 domain; for example, the D-box degron binds to a pocket situated between blades 1 and 7, whereas the KEN-box binds at the centre of the top surface of the wheel-like WD40 repeat domain ^70– 72^. Mutations, in the degron module on a substrate or the corresponding channel surface in the WD40 domain, that block substrate–co-activator interaction, render that substrate unavailable for ubiquitylation. The D-box– and the KEN-box–binding pockets/surfaces are evolutionally well conserved despite slight variations between Cdc20 and Cdh1 and those among species. Yet this may be a matchmaker mechanism, generating variable and dynamic affinities, by which degron module sequences on candidate substrates can be scanned and interrogated. As a consequence, if recognised as a genuine substrate, the degron specifically binds the WD40 surface with the correct affinity programmed by its degron sequence, which may determine processive or poor ubiquitylation of the substrate. The strength of interaction is likely to be regulated by environmental cues as it has been reported that the phosphorylation state around the degron region can influence the ubiquitylation of substrates (for example, Cdc6) ^73^. Too strong or too weak interaction is probably not good for ubiquitylation. However, some APC/C inhibitors such as Mes1 or Acm1 seem to use such excessive affinity on purpose to inhibit the APC/C ^74– 76^. It should be noted that Cdh1 has a broader substrate specificity than Cdc20. It is likely that traits on the WD40 domain are responsible for such specificity, although the underlying mechanism remains obscure.

## Phosphorylation regulates APC/C ^Cdc20^ and APC/C ^Cdh1^

The APC/C is “cell cycle–regulated”, which was very clearly described in original discovery papers ^5, 6^. Cdc20 binds and activates mitotically phosphorylated APC/C ^77– 82^. However, because many sites on the APC/C subunits are phosphorylated by cyclin-dependent kinase 1 (Cdk1) ^83, 84^, the sites of phosphorylation and their impacts have not been defined. Recently, the expression of recombinant APC/C and extensive site-directed mutagenesis of different subunits has uncovered the mechanism underlying the activation of the APC/C by Cdk1 phosphorylation ^85, 86^ and this has been confirmed by high-resolution EM studies ^87^ ( Figure 3). The model also supports the theory that Cdk1-dependent APC/C phosphorylation is the trigger for anaphase onset. In addition, the study highlights the importance of disordered loop domains of the APC/C subunits for dynamic regulation. Although Apc3 and Apc1 are clearly key subunits for phospho-regulation, other sites are also phosphorylated *in vitro* and *in vivo*. The roles of such phosphorylation remain elusive. Furthermore, how phosphorylation of the APC/C is regulated by phosphatases or how “teamwork” phosphorylation with other mitotic kinases (for example, polo-like kinase) is achieved for APC/C regulation requires elucidation.

Cdk1-dependent phosphorylation of Cdh1 is inhibitory, as shown in the late 1990s ^88, 89^, explaining the observation that Cdh1 action is repressed in mitosis and Cdc20 is the predominant co-activator. However, like Cdh1 phosphorylation, Cdk1-dependent phosphorylation of Cdc20 was shown to block an APC/C activation role through the C-box ^63^. In mitosis, protein phosphatases such as PP2A dephosphorylate and activate Cdc20 ^63^. Yet the situation is slightly more complicated as in mitosis the APC/C needs to be phosphorylated ( Figure 3) whereas Cdc20 needs to be dephosphorylated for the C-box–dependent activation of the APC/C. How can this conundrum be resolved? One mechanism seems to involve PP2A substrate specificity. PP2A complexes have an inherent preference for phosphothreonine over phosphoserine ^90– 93^. Notably, the key Cdk1 sites around the C-box of Cdc20 are threonine ^63^ whereas Cdk1-phosphorylation sites in Apc1 loop ^300^ are exclusively serine ^85– 87^. Thus, Cdc20 can be more efficiently dephosphorylated than the APC/C, allowing APC/C ^Cdc20^ complex formation during the correct window. It is unknown exactly how and which subfamilies of PP2A are involved in Cdc20 and APC/C dephosphorylation and whether other phosphatases such as PP1 are involved and, if so, how they are regulated. It should be noted that key Cdk1 sites of Cdh1 are serine; thus, Cdh1 dephosphorylation and subsequent Cdh1-dependent APC/C activation occur only after Cdk1 inactivation and subsequent activation of Cdk-counteracting phosphatases ^89, 94– 97^, which initiate mitotic exit. Cdh1 phosphorylation is also involved in its subcellular localisation, contributing to the spatiotemporal regulation of the APC/C ^98– 100^.

## APC/C activity can be inhibited at multiple levels

**Figure 4.**
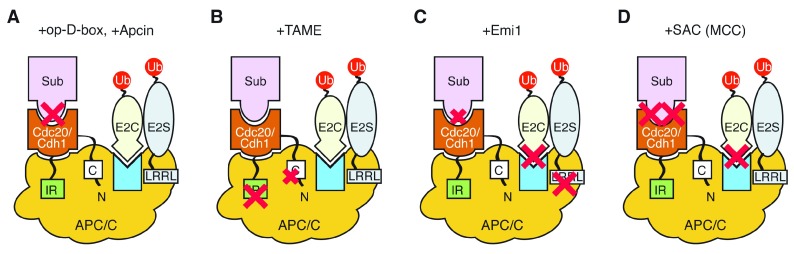
APC/C inhibitors target the APC/C at multiple levels. ( **A**) Overexpression or high dose of the destruction box (D-box) fragment (+op-D-box) competes with substrates to bind to the D-box–binding pocket on the WD40 domain (competitive inhibition). A small-molecule Apcin binds the D-box–binding pocket on the side face of the WD40 domain (+Apcin). ( **B**) A small-molecule tosyl- l-arginine methyl ester (TAME), which resembles the isoleucine-arginine (IR) tail of Cdc20 and Cdh1, binds APC3 to interfere with the IR tail–binding site (+TAME). EM study suggests that TAME might compete with Cdc20 to bind at the IR tail and the C-box–binding sites. ( **C**) Emi1 inhibits the APC/C at multiple levels (+Emi1). The D-box (weak) binds the WD40 domain of Cdc20/Cdh1, a zinc-binding region (ZBR) interferes with Ubc2C-dependent APC/C activity and the C-terminal LRRL tail interferes with Ube2S binding to the APC/C. The LRRL tail sequence of Emi1 is identical to the LRRL motif of Ube2S. *In vivo* target of Emi1 is Cdh1. ( **D**) The main effector of the spindle assembly checkpoint (SAC) is the mitotic checkpoint complex (MCC), which inhibits the APC/C at multiple levels. *In vivo* target of MCC is Cdc20. MCC binds both the D-box–binding pocket and the KEN-box–binding surface of the WD40 domain and blocks WD40-mediated substrate binding. MCC also blocks Ube2C-dependent APC/C activity at the closed MCC configuration; however, Ube2S-dependent APC/C activity is not inhibited by MCC. Schematic diagrams are based on the cartoon view of the APC/C in Figure 3 (bottom left). APC/C, anaphase-promoting complex/cyclosome.

Inhibitors are often very useful to explore the underlying mechanisms or processes of how a regulatory system works as critical processes are often targeted ( Figure 4). Classic APC/C inhibition may involve overproduction of the D-box (high dose of the D-box) fragments, which can overwhelm the substrate recognition of Cdc20 ( Figure 4A) and arrest cells at metaphase (by inhibiting APC/C) ^101– 103^. This finding suggested that proteins other than cyclin must be degraded to initiate anaphase, leading to the discovery of Cut2/securin ^12, 13^. Through the degron–WD40 interactions, Mes1 in *Schizosaccharomyces pombe* acts as a pseudo-substrate inhibitor for Fzr1/Mfr1 but works as a competitive substrate for Slp1/Cdc20, by which Mes1 controls the activity of the APC/C required for the meiosis I/II transition ^76, 104, 105^. Similarly, the degron motifs of Acm1 in *Saccharomyces cerevisiae* are recognised in different ways by the WD40 domain of Cdh1 and Cdc20 ^70, 74, 75, 106, 107^, so that Acm1 becomes an inhibitor of Cdh1 but not Cdc20.

Through a chemical genetic screen in *Xenopus* egg extracts, tosyl-L-arginine methyl ester (TAME), a small-molecule APC/C inhibitor, has been isolated ^108^. TAME structurally resembles the IR tail of co-activators and thus blocks Cdc20/Cdh1 loading onto the APC/C via the IR tail ^109^ ( Figure 4B). As the C-box–binding site on Apc8 is structurally equivalent to the IR tail–binding site on Apc3 ^32^, TAME potentially affects the C-box function as well as the IR tail ^87^. TAME is more specific to Cdc20 than Cdh1. This seems to be due in part to the fact that Cdh1 has more contact with the APC/C, achieving higher affinity, but the detailed mechanism remains elusive. Another inhibitor molecule, known as Apcin, which was isolated from the same chemical screenings, binds to the D-box–binding site of the WD40 domain of Cdc20 ( Figure 4A) ^110^. This finding has created a great opportunity for synergistic inhibition using both TAME and Apcin, which has proven to be more effective than either alone ^110^.

 Early mitotic inhibitor (Emi)1 is a metazoan APC/C inhibitor ^111^ which is a vertebrate homologue of Rca1 (regulator of cyclin A). In *Drosophila*, Rca1 inhibits APC/C ^Cdh1^ and stabilises cyclin A in S phase ^112, 113^. Emi1 has been shown to inhibit both APC/C ^Cdc20^ and APC/C ^Cdh1^ activity *in vitro* ^111, 114^ but its main purpose is thought to be inhibition of APC/C ^Cdh1^ during S and G _2_^115, 116^. ** Emi1 has a C-terminal inhibitory domain composed of structural components such as the D-box, Linker, ZBR and RL tail (Emi1 ^DLZT^) ^117, 118^. The Emi1 C-terminal domain was previously proposed to be a pseudo-substrate inhibitor ^119^, but cryo-EM and quantitative biochemical analysis have revealed a more sophisticated inhibition mechanism; Emi1 apparently uses every structural property within the Emi1 ^DLZT^ domain and blocks APC/C ubiquitylation processes, including Ube2S-dependent ubiquitin chain elongation ^32, 117, 118^ ( Figure 4C). It should be noted that Emi1 destruction is regulated by another E3 ubiquitin ligase, SCFβTRCP, through the degron on its N-terminal domain upon phosphorylation ^120– 122^. Also, Emi1 activity is negatively regulated by Cdk1 phosphorylation ^123^. Because of the high potency of Emi1, it is controlled by multiple layers of regulation, including transcriptional and translational changes ^124, 125^. Emi2 (also called Erp1), a maternal paralogue of Emi1, inhibits the APC/C in a similar manner to Emi1, so that vertebrate eggs awaiting fertilisation are arrested at metaphase of meiosis II ^126, 127^.

Finally, the spindle assembly checkpoint (SAC) monitors unattached or tensionless kinetochores and delays the onset of anaphase until all the kinetochores are attached to form a proper bipolar spindle structure ^128– 130^. The mitotic checkpoint complex (MCC) consisting of Mad2, BubR1, Bub3 and Cdc20 is a potent APC/C inhibitor ( Figure 4D). MCC was recently shown to inhibit a second Cdc20 ^APC/C^ that has already bound and activated the APC/C, highlighting that MCC can indeed act as a direct APC/C inhibitor, rather than sequestering Cdc20 ^131^. High-resolution cryo-EM studies of APC/C-MCC complex reveal that BubR1 binding mislocates Cdc20 ^APC/C^ and blocks substrate recognition ^48, 49^. The MCC docks into the APC/C central cavity and also interferes with Ube2C recruitment, inhibiting most of the substrate ubiquitylation. Intriguingly, a subset of substrates such as Nek2A or cyclin A, which can bind the APC/C independently of the WD40 of co-activators, can be degraded even when SAC is active ^132– 134^. It might be that Nek2A binds to a TPR subunit that MCC does not interfere with (for example, Apc6B or Apc7) by which Nek2A is ubiquitylated and degraded as long as the proper interaction between the C-box and Apc8B is ensured. Once all kinetochores become stably attached to the spindle, the SAC has to be silenced to allow anaphase onset. p31 ^comet^ is an SAC antagonist ^135^ and is involved in SAC silencing in multiple ways, such as blocking Mad2 activation by binding to C-Mad2 ^136, 137^ and assisting MCC disassembly together with the AAA+ ATPase TRIP13 ^138– 143^. Cdc20 auto-ubiquitylation is also involved in MCC disassembly ^144– 149^. Yet it appears that several pathways regulate SAC activation as well as SAC silencing *in vivo* ^150– 155^, so further work is necessary to elucidate the detailed mechanisms. Regulation of the SAC pathway has been reviewed by others ^20, 21, 156– 158^.

## Future perspectives

Recent progress on the resolution of cryo-EM is amazing, entailing near atomic resolution now and inevitable atomic resolution in the future. The MultiBac-based reconstitution pipeline of the whole APC/C complex allows construction of any mutation(s) in any subunit(s) at will, which provides an unprecedented opportunity to interrogate detailed dynamic regulation in physiological conditions such as *Xenopus* egg extracts together with the latest structural technologies, in combination with cell biological, genetic, biochemical, bioinformatics or mathematical modelling approaches. By combining genome editing and RNA interference, mammalian cell biology approaches will also provide unprecedented details of APC/C regulation. Yet we still face a number of outstanding questions. Why is the APC/C so large and why are so many subunits required for APC/C activity? Has evolution contributed to the enormous size and the complexity of the subunits? Are there any as-yet-unidentified subunit or sub-complex functions besides ubiquitylation? Is the APC/C complex disassembled partly or even fully and how is it regulated? We know that cellular subunit expression levels vary depending on subunit, so it may be that some subunits behave as a core regulating the assembly. Another key issue is the influence of subcellular localisation on APC/C function *in vivo*. Local concentration of not only the APC/C but also co-activators, E2s, substrates and inhibitors would all affect APC/C activity. Furthermore, our knowledge of ubiquitin dynamics regulating and maintaining the relationship between the APC/C and the action of DUBs is still very limited, although very recently Cezanne/OTUD7B was shown to be a cell cycle–regulated DUB antagonising APC/C activity ^159^. Moreover, increasing evidence suggests that dysregulation of Cdc20 or Cdh1 is involved in disease conditions and progression as in cancer. Deeper knowledge of the APC/C ubiquitin system and the mechanisms of distinct co-activator working will ultimately contribute to not only a better understanding of the cell cycle but also the possible development of therapies or tools to control or monitor dysregulated APC/C.

## Closing remarks

The discovery of MPF as a complex of cyclin B and Cdk1/Cdc2 in the late 1980s heralded the first wave of understanding of the cell cycle in modern times. Many scientists following their own interests and curiosity had conducted studies in a number of model organisms such as frog, starfish, clam, sea urchin, yeast (budding and fission), fly and human cell culture systems ^160– 173^. Collaborative and comparative analysis of all this research unveiled the MPF story. In September 1988, at a key moment in the beginning of cell cycle research, the first CNRS Cell Cycle meeting in Roscoff (France) was organised, highlighting the efficacy of collaboration and a multidisciplinary approach to solve important questions in science. With continued passion and curiosity, hard work and luck, much can be achieved, not only to further our understanding of the cell cycle but to pave the way for exciting new advances in the field of medicine.
